# Evaluation of the Virtual Multidisciplinary Team Meeting Model for Adult Patients on Haemodialysis: A Qualitative Study

**DOI:** 10.1111/jep.70071

**Published:** 2025-04-01

**Authors:** Qiumian Wang, Yangama Jokwiro, Edward Zimbudzi

**Affiliations:** ^1^ Department of Nephrology Monash Health Melbourne Australia; ^2^ Chronic and Complex Care Renal Services, Western Health Melbourne Australia; ^3^ La Trobe Rural Health School La Trobe University Melbourne Australia; ^4^ School of Nursing and Midwifery Monash University Melbourne Australia; ^5^ School of Public Health and Preventive Medicine Monash University Melbourne Australia

**Keywords:** COVID‐19, dialysis, multidisciplinary meeting, Nephology, virtual, VMDT

## Abstract

**Background:**

During the COVID‐19 pandemic, face‐to‐face multidisciplinary team meetings evolved to virtual platforms. Healthcare professionals' experiences of virtual multidisciplinary team meetings is unknown, and it is not clear whether virtual meetings are a feasible long‐term alternative in the post pandemic era.

**Objective:**

To explore the experiences and perceptions of members of the multidisciplinary team managing people with kidney disease regarding virtual meetings.

**Design:**

Semi‐structured interviews were conducted. Maximal variation sampling was used to ensure adequate representation by gender and professional roles. All interviews were audiotaped and transcribed verbatim, before being analysed by two researchers independently using the Theoretical Domains Framework. A third researcher was then referred to for resolving any disagreements.

**Participants:**

Members of the nephrology multidisciplinary team meeting.

**Measurements:**

Health professionals' experiences and perspectives of virtual multidisciplinary team meetings.

**Results:**

Of the nine participants interviewed, six were females and the majority were nurses. Most of the participants were aged 30–40 years. Three main themes emerged within the three primary domains: impact on staff and patient outcomes; limited technological skills, and opportunities for improvement. From the four intermediate domains, another four themes were captured: professional responsibility; impact on engagement; barriers to participation; and desire to provide optimal patient care.

**Conclusions:**

Healthcare professionals of a single centre nephrology care team reported that virtual multidisciplinary meetings overcame geographic barriers and infection control restrictions, and offered possibilities for broader inclusivity. However, strategies are needed to overcome technological issues, improve participants' skills to navigate technology, and optimize active participation.

## 1 Introduction

A multidisciplinary team (MDT) approach is an integrated model to healthcare in which medical, nursing, and allied health professionals of related specialties collaboratively discuss and develop the most suitable treatment plans for patients [1]. The MDT approach is widely accepted, studied and known to be effective in encouraging shared decision‐making, improving overall patient survival and patient satisfaction among people with chronic diseases [1, 2, 3, 4, 5]. For instance, among people with cancer, the MDT approach improved care coordination and adherence to evidence‐based treatment recommendations [6] while in nephrology, the approach improved patients' knowledge about chronic kidney disease, and dialysis modalities, which can potentially increase the proportion of dialysis starts in the outpatient setting, as opposed to inpatient setting.

Traditionally, MDT approach involved collaborative meetings [7] conducted face‐to‐face until when the COVID‐19 pandemic forced them to be switched to virtual platforms. Before the pandemic, the virtual MDT meetings were sporadically used to overcome geographic barriers associated with face‐to‐face meetings and reduce the opportunity cost of conducting MDT meetings regularly [8]. The sporadic use of virtual platforms has been associated with a number of disadvantages which include loss of richness of information delivered by non‐verbal cues and constrained effective communication [9]. Participants might adopt a more assertive communication style unconsciously in response to these disadvantages, which could inhibit collaborative discussion, thus negatively affecting patient care [8, 9, 10].

Given that virtual MDT meetings within the nephrology care are a contemporary approach, an urgent evaluation of these meetings is warranted for several reasons. First, healthcare professionals' experiences with virtual MDT meetings are unknown. Second, the impact of virtual MDT meetings on patient outcomes has not been studied. Lastly, there is need to establish whether virtual MDT meetings are a feasible long‐term alternative in the post‐pandemic era. In this qualitative study, we aimed to explore the experiences and perceptions of all members of the MDT managing people with kidney disease regarding virtual meetings, in the setting of a single adult acute dialysis unit affiliated to a large metropolitan teaching hospital situated in the south‐eastern region of Melbourne, Australia.

## 2 Methods

### 2.1 Design

This study utilized Framework Analysis, which is a comprehensive approach used for both inductive and deductive examination of qualitative data [11, 12]. The choice to employ the framework analysis methodology was driven by its proven advantages, especially when handling extensive homogeneous qualitative data [12]. In particular, the researchers utilized the Theoretical Domains Framework (TDF) which served as a structured guide and is commonly used in implementation research [13]. It comprises 14 distinct domains, each of which represents a collection of constructs that can encompass factors influencing a particular behavior [13]. These 14 domains are detailed in Table 1.

**Table 1:** TDF domains and descriptions.

Domain	Description
Knowledge	Existing procedural knowledge, knowledge about guidelines, knowledge about evidence, and how that influences what participants do
Skills	Competence and ability about the procedural techniques required to perform the behavior
Social/professional role and identity	Boundaries between professional groups (i.e., is the behavior something the participant is supposed to do or someone else's role?)
Beliefs about capabilities	Perceptions about competence and confidence in performing the behavior
Optimism	Whether participants' optimism or pessimism influences what they do
Beliefs about consequences	Perceptions about outcomes, advantages, and disadvantages of performing the behavior
Reinforcement	Previous experiences that have influenced whether or not the behavior is performed
Intention	A conscious decision to perform a behavior or a resolve to act in a certain way
Goals	Priorities, importance, commitment to a certain course of actions or behaviors
Memory, attention, and decision processes	Attention control, decision making, memory (i.e., is the target behavior problematic because participants simply forget?)
Environmental context and resources	How factors related to the setting in which the behavior is performed (e.g., people, organizational, cultural, political, physical, and financial factors) influence the behavior
Social influences	External influence from people or groups to perform or not perform the behavior; how the views of colleagues, other professions, patients and families, and doing what you are told influence the behavior
Emotion	How feelings or affect (positive or negative) may influence the behavior
Behavioral regulation	Ways of doing things that relate to pursuing and achieving desired goals, standards, or targets; strategies the participants have in place to help them perform the behavior; strategies the participants would like to have in place to help them

*Note:* Adapted from [14].

### 2.2 Participants Selection

The sampling pool included 22 regular nursing staff of the adult haemodialysis unit, a nephrologist, renal registrar, social worker, dietician, renal access coordinator, and a dialysis coordinator. They were purposively sampled to ensure a diverse range of views was captured. Maximal variation sampling was used to ensure adequate representation by gender and professional roles. Casual nursing staff and nursing students were excluded due to lack of active participation in the MDT meetings. All participants gave written consent before participating in the study.

### 2.3 Data Collection

The interview guide was developed by an experienced qualitative researcher (EZ, PhD, male) in consultation with a senior dialysis nurse (QW, MNNP, female) who participated in the virtual MDT meetings. To thoroughly examine the participants' experiences with the VMDT meetings, the interview guide included key questions focused on the frequency of their attendance, their perceptions of the meetings, and whether they believed their contributions impacted decision‐making processes. Additionally, the guide provided an opportunity for participants to reflect on the influence of the VMDT meetings on patient outcomes (Appendix A). To address the perception of coercion which may occur due to research being conducted by a colleague, the voluntary nature of participation was explained and potential participants were informed that they had the right to withdraw from the study at any stage without any repercussions. Participants were assured that their involvement in the research would not result in them being treated differently at work. Semi‐structured interviews were performed from 1st of March 2022 to 30th March 2023 amongst members of the MDT meeting to explore their experiences and perspectives of virtual MDT meetings. The interviews were conducted by a novice researcher, QW, under the guidance of a senior researcher, EZ. Interviews were conducted either face‐to‐face at the workplace or virtually on Microsoft Teams and Zoom platforms or phone call, depending on the participants' preference, until thematic saturation was achieved [15], meaning that additional data no longer provided new insights or contributed to the emergence of new themes. This indicated that the full range of relevant experiences and opinions related to the research question had been thoroughly captured. Each interview lasted between 20 min to 30 min. The interviews were audiotaped verbatim. No field notes were made during the interviews. No repeat interviews were carried out.

### 2.4 Data Analysis

Deidentified audiotaped interviews were transcribed verbatim and analysed following an inductive and deductive approach, utilizing the TDF as the analytical framework. Participant experiences and perceptions were aligned with the relevant domains of the TDF. Transcripts were not returned to participants for comment due to the application of a robust and structured analytical approach, specifically the Theoretical Domains Framework. Furthermore, because some transcripts contained sensitive information, the researchers determined that returning them to participants would pose a risk to confidentiality and could inadvertently expose private data. Coding commenced by reviewing the transcript for each participant's response, assessing connection to the definitions of the domains and/or the constructs within those domains. Subsequently, attributions were made to the respective domain. In instances where information could pertain to multiple domains, coding was applied to all pertinent domains, with a preference for attributing information to the most relevant domain. To identify the most pertinent theoretical domains, a systematic and rigorous process was employed, integrating both theoretical considerations and empirical data. Specifically, the relevant theoretical domains were determined based on the frequency of specific beliefs and themes, the presence of conflicting beliefs, and the identification of strongly held beliefs that may influence behaviors. Consensus of the emerging domains was reached between the two researchers (EZ and QW). Any disagreements were resolved by referring to the third researcher (YJ, PhD, male) for adjudication.

Following the coding of interview data into theoretical domains, a secondary analysis was carried out within these domains to identify subthemes. This subsequent analysis phase adopted a more inductive approach. Key belief statements were formulated to encapsulate the emerging subthemes or key messages within each domain. Additionally, they captured contextual factors influencing participants' attitudes toward VMDT meetings. The theoretical domains most pertinent to the analysis were determined by several criteria, including the frequency of mention by participants, the relatively larger number of identified subthemes, the extent of discussion, and the perceived passion conveyed by participants during the audiotaped discussions.

## 3 Results

Participants (*N* = 9) included 6 females and 3 males, and the majority were aged 30–40 years (56%) (Table 2). Most of the participants were nurses with a wide range of dialysis nursing experience. Themes and illustrative quotes are summarized in Appendix B.

**Table 2:** Demographic and professional roles of the participants.

Participants (*N* = 9)	*N* (Percentage)
**Demographics**	
Female	6 (67)
Age group	
20–30	1 (11)
30–40	5 (56)
40–50	1 (11)
50–60	2 (22)
**Professional roles**	
Registered nurse	7 (78)
Medical doctor	2 (22)

### 3.1 Primary Domains

The three predominant domains were “Beliefs about consequences”, “Skill”, and “Goals”. Three themes, containing two or three subthemes emerged from these domains. The themes and subthemes identified were 1) Impact on staff and patient outcomes (improving patient outcomes, improving accessibility, and providing inter/intra disciplinary learning opportunities); 2) Limited technological skills (technological challenges, lack of skills to adapt and navigate technology); 3) Opportunities for improvement (improvement in technology, improvement in inclusivity, and shared responsibility).

#### 3.1.1 Impact on Staff and Patient Outcomes

##### 3.1.1.1 Improving Patient Outcomes

Most participants believed that the VMDT meeting contributed to better patient outcomes by enabling individualized care, providing a platform where patient care issues can be discussed in a broader team, promoting problem‐solving collaboratively and avoiding variability between clinicians, and providing opportunities to discuss issues that cannot be addressed timely in a busy clinical environment.

It's huge value to the patients and has a huge impact on their outcomes and care because you've got those open discussions between all disciplines that are all working towards them as a patient and their problems as patients and getting the best out of their treatment while they under our care

—P2

However, one participant had mixed feelings regarding whether the VMDT meeting positively influenced patient outcomes.

Well, I'd hope that improves them. I don't know about that. But I think it does.

—P6

Participants also acknowledged that the VMDT meetings provide easy‐to‐follow meeting records and clear plan for patient care that can be followed up.

When I read the minutes, I know that, what are they planning to do with the patient. So, I can execute that later when I'm looking after the patients. So, it definitely has got positives

—P1

##### 3.1.1.2 Improving Accessibility

Participants identified that the VMDT meeting overcomes location barriers and makes it possible to attend without having to be present physically. It overcomes geographic barriers and COVID‐related infection control restrictions.

It's actually easy because wherever you are…… (you can) still listening to the virtual meeting, which makes it very handy. When you have to do it face to face, if you're not working, you don't have any option to attend the meeting. It's actually very convenient

—P8

##### 3.1.1.3 Providing Inter/Intra Disciplinary Learning Opportunities

Another benefit of the VMDT meeting identified by the participants is that it provides learning opportunities across different disciplines. It is recognized as a great tool for team building, teamwork, and team learning.

It's also really good for a learning perspective, when we're talking about the patient's problems, there's a bit of teaching that goes on too……. it gives you a bit of insight for us as nurses into the other disciplines, whether it be access nurse, medical, dietitian, whatever, and vice versa, around the table

—P2

#### 3.1.2 Limited Technological Skills

##### 3.1.2.1 Technological Challenges

Technological issues such as unreliable internet, device issues, background noise, connection issues, lagging and delaying were mentioned most by participants. Some participants also acknowledged that technological challenges make it difficult to attend the VMDT meetings.

…. sometimes there's lag, so you can't hear anything. All things delayed sometimes. You know the IT comes under the heading of technical issues, but there's like a lot of them (technical issues)

—P3

Basically, because of technology, sometimes we cannot rely so much on the internet, like, if the connection is not good, then that's one of the disadvantages of having these virtual meetings

—P8

##### 3.1.2.2 Lack of Skills to Adapt and Navigate Technology

Some participants observed that virtual meeting courtesy, which includes being on mute when not communicating was not always practised. Additionally, some VMDT participants demonstrated limited skills in adapting and navigating virtual technology.

Sometimes people don't mute their thing, don't realize they haven't muted themselves, and this happens with all meetings. So, there's like all random noise

—P3

It's always hard to find the link

—P3

#### 3.1.3 Opportunities for Improvement

##### 3.1.3.1 Improvement in Technology

When asked for suggestions to improve VMDT meetings, almost all participants mentioned technological improvements. Suggestions such as better sound, connection and equipment, platform standardization, and backup plan for technological issues were given.

(We need to improve) the technology side of things, with the issues around, the sound, the connection, and the computers…

—P2

If we are able to standardize across the different meetings in one platform, that would probably be ideal.

—P3

There should always be a backup plan if there is a communication problem or connection error, so the meetings don't get interrupted so much

—P4

##### 3.1.3.2 Improvement in Inclusivity

Suggestions for improvement also included extending the VMDT meetings to other relevant healthcare professionals outside of the unit for inclusivity, which would benefit patient care.

…. hope (someone) from a transient or in a satellite unit (can present)

—P5

##### 3.1.3.3 Shared Responsibility

Furthermore, many participants acknowledged that responsibilities often fall only on members who actively participate in VMDT meetings. This led to suggestions such as giving specific tasks and regular evaluations to ensure the quality of meeting participation, encouraging active meeting participation, and shared responsibility.

……from each (nursing) group, if someone who is also given the responsibility to present (the patients), it would be better……. For example, the group members who are actually preparing the list, and sending it to our manager.

—P1

There could be more active participation from all the participants, not just the regular participants.

—P5

### 3.2 Intermediate Domains

Domains less frequently mentioned included “Social/professional role and identity”, “Reinforcement”, “Environmental context and resources”, and “Behavioral regulation”. Four themes emerged from these domains and they were (1) Professional responsibility; (2) Impact on engagement (loss of face‐to‐face interaction); (3) Barriers to participation; (4) Desire to provide optimal patient care.

#### 3.2.1 Professional Responsibility

Some participants attributed their active VMDT meeting participation to their professional role, portfolio, or responsibility.

In my experience, I had to really be like a very active participant of it because of my role in the unit. So, with how our meeting works, we are divided into groups. And most of the time, it's either, I'm the group leader or I'm going to prepare the list, so, with that experience, I sort of handled most of our group's participation in the meeting.

—P4

However, poor participation was raised often as one of the shortcomings of VMDT meetings and reasons for this included lack of accountability, leaving responsibility to others, multi‐tasking, and less prioritization.

Just observing what was happening during some of the online meetings, people were multitasking and maybe flicking between doing notes and then listening to meetings, which probably wasn't as beneficial as maybe it could have been.

—P2

#### 3.2.2 Impact on Engagement

##### 3.2.2.1 Loss of Face‐to‐Face Interaction

Most participants raised the issues of loss of face‐to‐face interaction in VMDT meetings compared to face‐to‐face MDT meetings, which negatively impacted on meeting engagement.

When it comes to face‐to‐face, it's much easier because we're forced to go into a meeting room, people are sitting together, everyone gets a chance to say what they have to say. They don't have to wait for others to finish. Or they don't think—why should I talk?

—P5

#### 3.2.3 Barriers to Participation

In terms of barriers to meeting attendance, location, work schedule and other obligations, timing of the shift and other commitments were identified.

If I'm not here (at work), I fail to log on from home, if I'm working here, but my patients come off (dialysis), or I might be busy doing something like handing over the patient, (I also can't attend)

—P1

It's more likely I will attend if I'm working in the morning, because if it's in the afternoon, it impacts my work

—P4

#### 3.2.4 Desire to Provide Optimal Patient Care

In terms of enablers to meeting attendance, thriving for best patient care and involvement in patient care were identified.

What makes me attend more is my motivation to improve patient care, and to follow up care for a patient.

—P7

There are a lot of issues going on within the dialysis unit, that does not only pertain to my patients, but to other patients, and I've got a lot of involvement with those incidents and cases as well, so I would attend (the VMDT meetings)

—P4

### 3.3 Infrequent Domains

The seven least frequently discussed domains included “Knowledge”, “Beliefs about capabilities”, “Optimism”, “Intention”, “Memory, attention, and decision processes”, “Social influences”, and “Emotion”.

## 4 Discussion

The purpose of this study was to explore the experiences and perceptions of all members of the MDT managing people with kidney disease regarding virtual meetings, in the setting of a single adult acute dialysis unit affiliated to a large metropolitan teaching hospital in Australia. Three main themes emerged within the three primary TDF domains: impact on staff and patient outcomes; limited technological skills, and opportunities for improvement. From the four intermediate TDF domains, another four themes were captured: professional responsibility; impact on engagement; barriers to participation; and desire to provide optimal patient care.

One of the most important findings in this study is that the VMDT meetings overcome geographic barriers and COVID‐related infection control restrictions. This is consistent with findings from several studies [16, 17, 18, 19, 20, 21, 22, 23, 24, 25]. This is valuable because VMDT meetings ensure continuity of patient care in situations when face‐to‐face meetings cannot take place thereby allowing benefits associated with MDT meetings to continue. Furthermore, looking beyond the COVID‐19 pandemic, some participants in this study also found the virtual platform more convenient than face‐to‐face delivery because it enabled participation despite work schedules and physical locations. This finding is similar to the results reported by Groothuizen et al. [16] and Bonanno et al. [22]. Bonanno et al. [22] also pointed out that such an advantage of the VMDT meetings may explain their findings of increased MDT meeting participation rate.

Additionally, participants in this study also suggested that VMDT meetings could be more inclusive by inviting healthcare professionals who are outside of the immediate nephrology team but are involved in patient care. This is an important suggestion given that almost all people with advanced chronic kidney disease have comorbidities and concordant diseases, such as heart failure, diabetes, pain, and mental health conditions such as depression, schizophrenia or bipolar affective disease [26]. Having other health professionals such as diabetes educators, heart failure experts, pain specialists, and hospital or community mental health teams in the MDT meetings would be invaluable in providing holistic care for individual patients, as well as interdisciplinary learning. Understandably, MDT meetings with people from different teams across different healthcare settings are difficult to organize due to geographic barriers. However, the virtual platform removes such barriers and offers an exciting opportunity for inclusivity. Such VMDT model across different specialities has been reported in cancer care [17, 18], but to the authors' knowledge, it has not been in practice in nephrology care. Perhaps it is time to take this opportunity to optimize the MDT meetings. Admittedly, including healthcare professionals outside of the immediate nephrology team poses its own challenges such as scheduling conflicts. Relationship building based on mutual patient care before inviting other health professionals is vital. Dedicating certain part of the VMDT to discuss mutual patient care to save time for other health professionals, clear agenda/discussion points, effective discussion facilitation, and active participation are all important to achieve inclusivity.

One of the major shortcomings of the VMDT meetings perceived by participants is technological limitations which are: lack capability of IT infrastructure and lack of skills to navigate technology. The reported lack of capability of IT infrastructure includes unreliable internet, device issues, connection issues, lagging and delaying are consistent with previous studies [16, 20]. Some participants in our study even identified IT infrastructure issues as barriers of attending VMDT meetings. Our study, like most previous studies, calls for IT infrastructure improvements and real‐time IT support to ensure meeting accessibility, saving valuable clinician time, and smooth discussion. Many reported technological issues in our study, however, are not related to IT issues, which include background noises during VMDT meetings, difficulty of finding meeting links and difficulty with equipment. This can be attributed to lack of skills to navigate technology and lack of virtual meeting courtesy. Adequate training and regulations are needed to overcome this issue and ensure meeting quality. Kerawala et al. [27] suggested using “user's guide for IT issues, or even an IT “dummy run” for practice to upskill participants and familiarize participants to the meeting platforms. Another approach is for nephrology units to create educational competency packages tailored to VMDT, which should be accessible to all new staff and completed on an annual basis. Some members of the nephrology teams can also be trained in VMDT to become champions, a strategy that has proven effective in the successful implementation of technology in healthcare services [28]. Gross et al. [29] also proposed regulations for VMDT courtesy, such as “communication hygiene”, which is one participant speaking at one time, having a moderator, as well as all participants having cameras on and staying unmute. However, our study found that it is preferable to stay muted when not speaking, to avoid background noises and echoes. This is consistent with findings from Bonanno et al. [22].

An additional perceived shortcoming is lack of active participation in the VMDT meetings, especially when compared to the traditional face‐to‐face MDT meetings. Loss of face‐to‐face interaction in VMDT meetings has been raised by most participants, which negatively impacted on meeting engagement. Possible reasons for this include participants not having cameras on, lack of accountability, multi‐tasking, and less prioritization. Our brains process in‐person and virtual interactions differently, with lower brain activities, less concentration, and more prone to distractions shown in virtual interactions [30]. It is far easier to yield to the temptation of continuing the tasks at hand, such as completing documentation or replying to emails, rather than prioritizing the VMDT meeting when people are facing a screen, especially when not being watched. This is similar to what Kerawala et al. [27] described as “human factors”. Possible strategies such as keeping all participants visible at all times, suitable meeting environment to minimize distraction, and assigning specific tasks to MDT members to ensure accountability may be helpful to facilitate active VMDT meeting participation. On the other hand, this study also found that professional roles within the healthcare team and desire to improve patient care are facilitators of active participation. Individuals who have more clinical responsibilities, are more involved in patient care, or have strong desire to improve patient care would attend the VMDT meetings more, as well as contribute more. This finding prompted a unique way to enhance participation in the VMDT meeting, by leveraging individuals' clinical responsibilities and desire to improve patient care. For example, clinical responsibilities can be divided, delegated and assigned to individuals with low participation; education, team building, and professional development activities can be organized to nurture professional pride and desire to improve patient care.

### 4.1 Limitations and Strength

This study has several imitations. First, interviews were conducted by a researcher who is also a member of the VMDT. Participants could have felt obligated to provide a socially acceptable response. However, participants were informed that their responses would be treated with confidentiality before the interviews to mitigate this effect. Second, other key members of the VMDT such as allied health professionals did not participate in this study. Their perceptions and experiences of the VMDT were not considered in this study. Third, our results may apply to VMDTs for people with kidney disease and not other conditions because this study was targeted at a single healthcare team within a specific department. Lastly, transcripts were not returned to participants for comment or validation due to clinical workload and time constraints, which may affect the accuracy of the data. In terms of study strength, this study was conducted concurrent to the practice of the VMDT meeting, therefore recall bias was minimized. Moreover, a rigorous analysis approach and a validated framework guided the conduct of this study.

### 4.2 Clinical Implications

This study has a number of clinical implications. In an era when technological advancement is becoming more rapid even in the healthcare settings, the findings of this study highlighted the necessity to formally embrace the ability of virtual meeting technologies to enhance communication and collaboration while overcoming geographic barriers and infection control restrictions through supportive guidelines, resources, training, and technical support. It is also necessary to have policies that recognize virtual meetings as formal substitutes to face‐to‐face meetings for employees to maintain professional levels of courtesy, confidentiality, and productivity. Additionally, policies should recognize the need for continuous improvement for innovative technologies and direct future research on the comparison of the effectiveness of the virtual MDT meetings when compared with traditional face‐to‐face meetings.

## 5 Conclusion

Health professionals of the MDT meeting managing people with kidney disease acknowledged that the biggest advantage of virtual meetings is overcoming geographic barriers and infection control restrictions, therefore enabling the MDT meetings to continue despite the COVID pandemic and offering possibilities for broader inclusivity. However, technological issues, lack of skills to navigate technology, and lack of active participation were identified as the main shortcomings of the VMDT meetings. Strategies such IT infrastructure improvement, adequate training, real‐time IT support, and online meeting courtesy reinforcement can be employed to overcome such shortcomings.

## Author Contributions

Study design: Qiumian Wang and Edward Zimbudzi. Data collection: Qiumian Wang. Data analysis: Qiumian Wang, Yangama Jokwiro, and Edward Zimbudzi. Study supervision: Edward Zimbudzi. Manuscript writing: Qiumian Wang. Critical revisions for important intellectual content: Yangama Jokwiro and Edward Zimbudzi.

## Ethics Statement

This study was approved by Monash Health Human Research Ethics Committee, ERM Reference Number: HREC/79857/MonH‐2021‐283000(v1).

## Conflicts of Interest

The authors declare no conflicts of interest.

## Data Availability

The data that support the findings of this study are available on request from the corresponding author. The data are not publicly available due to privacy or ethical restrictions.

## Appendix A Virtual MDT Meeting Study Interview Guide V1 15/02/2022

### A.1

To get started, I would like you to think about your experience of the incentre dialysis unit Virtual Multidisciplinary Team meeting (VMDT).

− May you tell me a bit more about your experience of the VMDT?
  Probe: What is your opinion/perception of VMDT?
− Of the last 10 VMDT meetings, how many have you attended?
− What could have influenced your attendance?
− Do you feel that your contributions influence decisions made during the VMDT?
− If not, what do you suggest needs to be done for you to be heard?
− In your opinion, what are the benefits of this meeting?
− What are the shortcomings of the VMDT?
− In what ways does the VMDT influence patient outcomes?
− What would you like to be done to optimize the benefits of this meeting?
− The VMDT has a number of healthcare professionals from different disciplines. Is there any representation that you think need to be included?
− In closing, is there anything you would like to let me know about the VMDT?

## Appendix B

### B.1

See Table B1.

**Table B1:** Themes and illustrative quotes.

Predominant domain	Theme	Subtheme	Quotation
Beliefs about consequences	Impact on staff and patient outcomes	Improving patient outcomes	“It's huge value to the patients, and has a huge impact on their outcomes and care, because you've got those open discussions between all disciplines that are all working towards them as a patient and their problems as patients and getting the best out of their treatment while they under our care”
			—P2
			“Patients' issues are being talked about and solutions are being provided. And if solutions are not provided on the spot, plans are being made for that”
			—P4
			“(issue) isn't properly addressed, get a chance to be addressed properly”; “We collectively go through the patient, follow the patient previous results of the previous treatment and we collectively look into the problem and see what happens. So instead of different people put their input in different times, we are collectively putting the input in one time looking at what others have done in different times. Yeah. That I think in turn translate into a better patient outcome”
			—P5
			“I know what they planning to do with the patients (from the meeting). So I can execute that later when I'm looking after the patients. It definitely has got positives (impact on patient care), yes I believe”
			—P1
			“Everyone can contribute to what's needed……. Patient's problems are presented, everyone gets to say what they think. So it's good for the patient”
			—P6
		Improving accessibility/geographic barriers/infection control	“It's actually easy because wherever you are…… (you can) still listening to the virtual meeting, which makes it very handy. When you have to do it face to face, if you're not working, you don't have any option to attend the meeting. It's actually very convenient”
			—P8
			“I think it's more beneficial in the sense that we all have to be in different areas because we cannot congregate in the same room in the setting of a pandemic. Also if you're not able to come in person, you might still be able to join in”
			—P4
			“It's actually easily accessible, considering that the link to the patient virtual meeting is emailed to everyone. It's very handy, easily accessible”
			—P9
			“Overcome COVID restrictions (social distancing)”
			—P7
		Providing learning opportunities (inter/intra disciplinary)	“It's also really good for a learning perspective, when we're talking about the patient's problems, there's a bit of teaching that goes on too……. it gives you a bit of insight for us as nurses into the other disciplines, whether it be access nurse, medical, dietitian, whatever, and vice versa, around the table”
			—P2
			“It's good for education as well for all team members”
			—P6
			“I think it's a really good opportunity to interact with the entire haemodialysis unit and to learn about what the common problems are and how to deal with them:
			—P3
Skill	Limited technological skills	Technological challenges	“…sometimes there's lag, so you can't hear anything. All things delayed sometimes. You know the IT comes under the heading of technical issues, but there's like a lot of them (technical issues)”
			—P3
			“And the connection problems as well”
			—P4
			“It's not going smoothly or saving time because of technical issues, and access to computers, that sort of stuff”
			—P5
			“I think it's always plagued by technological issues. Never smooth.”
			—P6
			“Shortcoming would definitely be technical issues. Like sometimes there are microphone issues.”
			—P7
			“Basically, because of technology, sometimes we cannot rely so much on the internet, like, if the connection is not good, then that's one of the disadvantages of having these virtual meetings”
			—P8
		Lack of skills to adapt and navigate technology	“Sometimes people don't mute their thing, don't realize they haven't muted themselves, and this happens with all meetings. So there's like all random noise”
			—P3
			“it's always hard to find the link”
			—P3
			“The microphone is only effective when I'm using my iPhone with my own speaker, but the computer doesn't (work)…So it's frustrating in a way”
			—P7
Goals	Opportunities for improvement	Improvement in technology	“(We need to improve) the technology side of things, with the issues around, the sound, the connection, and the computers…”
			—P2
			“If we are able to standardize across the different meetings in one platform, that would probably be ideal.”
			—P3
			“So, probably improving the technology (is what needs to be done to optimize the benefit of the VMDT meetings)”
			—P6
			“There should always be a backup plan if there is a communication problem or connection error, so the meetings don't get interrupted so much”
			—P4
		Improvement in inclusivity	“…hope (someone) from a transient or in a satellite unit (can present)”
			—P5
			“I guess encouraging staff to actually really diligently attend it (VMDT meeting), especially if a certain staff or just like myself would want to know what's been happening with the patients”
			—P9
		Shared responsibility	“……from each (nursing) group, if someone who is also given the responsibility to present (the patients), it would be better……. For example, the group members who are actually preparing the list, and sending it to our manager.”
			—P1
			“There could be more active participation from all the participants, not just the regular participants.”
			—P5
			“Probably active participation as much as possible from all members of each group. I think we are improving. A lot of our members are part timers. This is a bit of a challenge.…… But I think we really have to work on it. We have to improve it. There's room to improve, but I think we're getting there.”
			—P7
Social/professional role and identity	Professional responsibility		“In my experience, I had to really be like a very active participant of it because of my role in the unit. So with how our meeting works, we are divided into groups. And most of the time, it's either, I'm the group leader or I'm going to prepare the list, so, with that experience, I sort of handled most of our group's participation in the meeting.”
			—P4
			“It's one of my portfolios…… And I'll be doing the minutes most of the times. I feel like I'm contributing into something”
			—P5
			“…when I'm attending the meeting, maybe I'm not fully concentrating on that… as an individual, maybe I'm doing something else, I'm just listening to it. Because it's a virtual meeting, and no one is actually looking (watching). Sometimes someone might be logged in, but they are not actually listening to it properly. Virtually when we are attending, it might not be a full participation from the other members (who) are actually on the screen”
			—P1
			“Just observing what was happening during some of the online meetings, people were multitasking and maybe flicking between doing notes and then listening to meetings, which probably wasn't as beneficial as maybe it could have been.”
			—P2
			“I find that anything virtual sort of loses people's attention, quite a lot. People don't pay attention as much, because if you do it in person, you're less likely to see a person walking around trying to set up a machine while we're (having a meeting), whereas when you're doing a virtual meeting, someone is most likely to multitask and not really pay attention to what's being talked about.”
			—P4
Reinforcement	Impact on engagement (negative)	Loss of face‐to‐face interaction	“I think if it's like a face‐to‐face, I think that active participation will be there.”
			—P1
			“I'm glad that we are maybe getting back to more face‐to‐face meetings. Because I just think that's more personable, I suppose, and offers a bit more chance to be involved.”
			—P2
			“When it comes to face‐to‐face, it's much easier because we're forced to go into a meeting room, people are sitting together, everyone gets a chance to say what they have to say. They don't have to wait for others to finish. Or they don't think—why should I talk?”
			—P5
			“One on one interaction is better when it's person to person. Half of the time I'm not quite sure who are talking because I can't see you.…. I think it's (not ideal)…. and people don't have the ability to ask questions easily (in virtual meetings).”
			—P6
Environmental context and resources	Barriers to participate		“If I'm not here (at work), I fail to log on from home, if I'm working here, but my patients come off (dialysis), or I might be busy doing something like handing over the patient, (I also can't attend)”
			—P1
			“It (attendance) depends on whether I'm working at the time, or doing other things, or on holidays etc.”
			—P3
			“It's more likely I will attend if I'm working in the morning, because if it's in the afternoon, it impacts my work”
			—P4
			“Attend most of the VMDT meetings because I usually work on Tuesdays (meeting date)”
			—P5
			“Every time we have a meeting, I'm working with my other job, so it's really so hard to (attend), even if I wanted to.”
			—P8
Behavioral regulation	Desire to provide optimal patient care		“I'm interested to know what's happening with the (patient) management decision making”
			—P5
			“What makes me attend more is my motivation to improve patient care, and to follow up care for a patient.”
			—P7
			“There are a lot of issues going on within the dialysis unit, that does not only pertain to my patients, but to other patients, and I've got a lot of involvement with those incidents and cases as well, so I would attend (the VMDT meetings)”
			—P4
